# “Better than my professor?” How to develop artificial intelligence tools for higher education

**DOI:** 10.3389/frai.2024.1329605

**Published:** 2024-04-10

**Authors:** Stefano Triberti, Raffaele Di Fuccio, Chiara Scuotto, Emanuele Marsico, Pierpaolo Limone

**Affiliations:** ^1^Department of Psychology and Education, Università Telematica Pegaso, Naples, Italy; ^2^Department of Humanistic Studies, University of Foggia, Foggia, Italy

**Keywords:** artificial intelligence, higher education, learning processes, intelligent tutoring systems, chatbot, conversational pedagogical agents, distance education

## Abstract

Artificial Intelligence (AI) tools are currently designed and tested in many fields to improve humans’ ability to make decisions. One of these fields is higher education. For example, AI-based chatbots (“conversational pedagogical agents”) could engage in conversations with students in order to provide timely feedback and responses to questions while the learning process is taking place and to collect data to personalize the delivery of course materials. However, many existent tools are able to perform tasks that human professionals (educators, tutors, professors) could perform, just in a timelier manner. While discussing the possible implementation of AI-based tools in our university’s educational programs, we reviewed the current literature and identified a number of capabilities that future AI solutions may feature, in order to improve higher education processes, with a focus on distance higher education. Specifically, we suggest that innovative tools could influence the methodologies by which students approach learning; facilitate connections and information attainment beyond course materials; support the communication with the professor; and, draw from motivation theories to foster learning engagement, in a personalized manner. Future research should explore high-level opportunities represented by AI for higher education, including their effects on learning outcomes and the quality of the learning experience as a whole.

## 1 Introduction

Artificial Intelligence could be defined as a technology able to perform tasks and activities that would be commonly regarded as typical of human cognition and reasoning. From the point of view of applied mathematics, the most well-known subset of AI is Machine Learning, namely algorithms that allow computers to learn from data, examples, and experience, rather than following pre-programmed rules (Sharma et al., 2019). However, AI is a multidisciplinary field (if not a set of several fields), encompassing philosophy, cognitive science, and social sciences focused on AI implementation in real-life contexts (Poola, 2017; Yin et al., 2021).

The implementation of AI in real-life environments requires a high-level understanding of the contexts and agents involved, as well as of the potential users’ expectations. Indeed, as the vast literature on technology acceptance shows, users’ intentions toward technology are determined by users’ attitudes and preconceptions rather than by technology’s actual effectiveness (Marangunić and Granić, 2015).

The use of AI in different contexts, such as education, has generated many concerns within the population. Studies show that although explicit attitudes are generally positive toward the use of AI, there is still resistance at an implicit level (Sebri et al., 2020; Fietta et al., 2021; Fortuna and Gorbaniuk, 2022; Triberti et al., 2023). In general, people working in the numerous fields that involve (or could involve) AI technologies show both an explicit and implicit positive attitude toward AI (Fietta et al., 2021; Schepman and Rodway, 2023). Regarding attitudes toward common-use tools such as ChatGPT, studies have shown that it is influenced not only by individual characteristics such as gender but also by previous experiences of use (Yilmaz et al., 2023). It is therefore necessary to provide clear knowledge regarding the capabilities of AI and to train responsible citizens and students in the appropriate use of technology. AI should be able to improve the educational process, combining human capabilities with technological resources.

On these bases, in the present paper we performed a purpose-oriented literature search of contributions to provide the reader with relevant information about the implementation of AI tools in higher education, with specific focuses on conversational pedagogical agents and the context of distance learning. While this is not intended to be a comprehensive representation of the field, it sets the foundation for presenting our viewpoint based on the implementation of similar tools in our university’s educational programs.

## 2 AI and the educational context: a focus on conversational pedagogical agents

The fast development of computer science witnessed over the past decades, led to a remarkable evolution of the computational paradigms designed to process a particularly large amount of information in various fields of application. In this sense, the educational context is not unfamiliar to this development, as shown by the advancements in the field of Artificial Intelligence in Education (AIED), which deals with studying the applications of AI in instruction, according to different perspectives and pedagogical paradigms (Tang et al., 2021; Zhang and Aslan, 2021).

To analyze the directions of this field of study, we need to clarify the role played by AI in the educational context through three main modalities characterized by different AI-learner relationships. The first modality sees the AI as an educational agent that *directs* the learning processes of the student. The second approach gives AI a *supporting* role in learning processes but in this case, the human-machine interaction emerges as an equal relationship aimed at solving an educational task. Finally, a third paradigm configures AI as an *extension* of the student’s intellectual abilities assigning the leading role to the student (Ouyang and Jiao, 2021).

A review conducted by Zhang and Aslan (2021) identified the main application modalities of AI in education in the form of Chatbots (conversational pedagogical agents) (Fryer et al., 2017), Expert Systems (Dias et al., 2015; Hwang et al., 2020a,b), Intelligent Tutoring Systems (Matsuda et al., 2020), Machine Learning (Wei et al., 2018; Arpaci, 2019), Personalized Learning Systems (Walkington and Bernacki, 2019), Visualizations (Keshav et al., 2017).

Conversational agents are a great example of the ways AI could contribute to education. Indeed, AI tools such as conversational pedagogical agents have the same characteristics of interactive learning environments such as appropriability, evocativeness and integration (Harel and Papert, 1990; Rospigliosi, 2023). Conversational agents are characterized by appropriability because users, interacting with applications such as ChatGPT, can obtain personalized answers to the questions they ask. Evocation, on the other hand, regards the ability of these tools to stimulate personal reflection by supporting the exchange of conversational questions and answers. Finally, this conversational approach allows for greater integration with existing knowledge and promotes a deeper understanding of multiple meanings and concepts. Conversational pedagogical agents can be used as auxiliary tools in training processes such as knowledge management, needs analysis, training organization and feedback on results. It has been shown that conversational pedagogical agents effectively support scaffolding (i.e., the incremental mastery of a concept) by meaningful interaction (Forsyth et al., 2020). Indeed, these tools support personalized training that can improve the quality of learning (Chen F., 2022; Chen Z., 2022).

The common purpose of the various branches belonging to this research field consists in the structuring of high-quality learning environments that can adapt promptly to the needs of the student, resulting in high-level educational outcomes (Tahiru, 2021). Considering the integration with other AI resources employed in education, a review by Zawacki-Richter et al. (2019) highlighted the main applications aimed at developing predictive models of student performance, streamlining evaluation processes, and personalization of educational paths. These objectives are met by applying computational techniques to automate tasks such as homework assignments, administrative tasks, student admission processes (Johnson, 2019) and information re-processing (Florea and Radu, 2019). More, these strategies can facilitate e-learning through the implementation of human-machine interactions and between users within the same educational platform (Zhang and Aslan, 2021); such interaction can reach particularly complex levels through integration with digital interfaces for the creation of Embodied Conversational Agents (ECA) that show human-like characteristics based on parameters such as non-verbal communication (Kay, 2012; Tahiru, 2021).

Nowadays, however, there are several important open questions related to AIED. An important part of the literature focuses on the digital infrastructure without considering the pedagogical strategies to facilitate the inclusion of these technologies in the educational environment (Chen L. et al., 2020; Chen X. et al., 2020). Moreover, the prototypes may present features that make their use difficult within real-world contexts (for example, the time and the skills that should be developed to use the prototypes do not fit well among the constraints of a university course) (Kabudi et al., 2021). It is particularly important to establish a fruitful collaboration between programmers and educators to apply AI in various educational contexts, without creating discomfort and disorientation in learners, in such a way that students and teachers could be trained in the informed use of similar technologies (Zhang and Aslan, 2021).

One of the strengths of AI lies in its versatility, as it can be easily integrated with various tools and technologies. This adaptability makes AI a potential game-changer in education, particularly in the realm of e-learning. The primary objective of AI in instruction is to provide support that is more effective to students through personalized learning paths tailored to their specific needs, whether in online or in-person settings.

## 3 Examples and important concepts for conversational pedagogical agents

One of the most significant functions of AI in the field of education is to recreate personalized, interactive and experiential learning environments (Peredo et al., 2011; Chassignol et al., 2018; Hwang and Tu, 2021) capable of engaging students by improving their learning skills (Mikropoulos and Natsis, 2011). For example, Hwang et al. (2020a) tested an expert system that adapted learning materials according to students’ affective state (i.e., anxiety toward math), and demonstrated that both problematic affective states and cognitive load were lower in the experimental group, which also obtained better learning outcomes. Indeed, it is important to consider the effects of AI use in education concerning cognitive load and its boundaries. According to a learning-focused conception of the construct (Paas et al., 2016), cognitive load could be intrinsic (related to the elaboration of learning materials), but also extraneous (related to additional activities within the learning process, e.g., searching for more information) or germane (related to improving the learning process itself, e.g., schema acquisition and automation). Theoretically, the cognitive load related to interaction with a system would interfere with learning depending on how much it could be configured as extraneous. In other words, a system designed to support learning would be helpful insofar as it facilitates or structures the delivery of learning materials or supports schema acquisition. On the contrary, a tool that requires additional effort to be understood and used would generate an extraneous cognitive load that hinders the learning process and possibly reduces its perceived quality.

It has been shown that chatbots, i.e., online software with conversational capabilities, improved students’ learning experience thanks to the personalization of content (Rus et al., 2013; Pokrivcakova, 2019). Chatbots with “humanoid” characteristics were found to be more effective in engaging students (Saerbeck et al., 2010; Olney et al., 2013; Johnson and Lester, 2016). However, it has been verified that exposure to chatbots with overly “human” features can cause a feeling of discomfort in the user that is referred to as the “Uncanny Valley effect” (Hanson et al., 2005; Ciechanowski et al., 2019; Song and Shin, 2024). For obvious reasons, many studies on chatbots and learning focused on language learning, with inconsistent results. Indeed, the review by Huang et al. (2022) identified some opportunities and roles that a chatbot could play within language learning processes (i.e., interlocutors, simulations, transmission, helpline, and recommendation) but also warned the readers about risks and shortcomings. Specifically, chatbots may sound unnatural due to technological limitations; secondarily, their positive effect on motivation may disappear over time due to the “novelty effect”; finally, interaction with chatbots could request divided attention and generate extraneous cognitive load that would negatively affect the learning process.

Consistently, Fryer et al. (2019) showed that learners preferred human to chatbot interlocutors, and interest in chatbots was predicted by pre-existent attitudes toward them, for example in terms of perceived utility. In a recent study (Haryanto and Ali, 2019), the authors used a smartphone application called Siri, capable of helping people acquire knowledge as it can respond in a personalized way to questions. The students who participated in the study welcomed this application with motivation and enthusiasm. Instead, another research (To et al., 2021) assessed the feasibility and effectiveness of a chatbot that gave users reminders, feedback and information to increase physical activity; while the tool obtained positive results in the experimental group compared with controls, its acceptance among participants was just moderate and only 35% of the sample expressed interest in continuing to use the chatbot.

Another category regards Web-based AI systems not based on chatbots but that could be integrated with them. These solutions track and store the interactions between the student and the system to acquire information about the user’s knowledge state, choice, learning style and other relevant attributes. These systems have been found effective in supporting learning and problem-solving processes as well as collaborative learning and peer interactions (Kahraman et al., 2010; Peredo et al., 2011). These systems can build personalized teaching environments adapted to the individual characteristics of the students (Kahraman et al., 2010). It has been verified that these systems can create teaching environments that present diversified content to students based on their previously assessed learning style (e.g., visual vs. verbal; preference for abstract concepts and generalizations vs. concrete practical examples) (Popescu, 2010). This approach increased the efficiency of the student’s learning process as the personalized teaching environment made it possible to acquire knowledge in a short time. Matching learning materials to the student’s learning style can reduce learning effort while increasing motivation and satisfaction. Such systems are still based on AI tools and could be effectively integrated with highly interactive interfaces that feature conversational pedagogical agents that adapt their communicational style based on user data collection (Baylor and Kim, 2005; Fryer et al., 2019; Bolarinwa et al., 2023); communicational style (e.g., formal or informal) could also impact students’ performance, motivation and perceived difficulty of tasks (Li and Graesser, 2017, 2021).

## 4 Experiences and reflections

In recent years, artificial intelligence technologies have supported teaching and learning processes in different ways, especially in the field of distance education (Dogan et al., 2023). In particular, the processes of educational data mining (EDM), i.e., the analysis of all the educational data recorded on the individual characteristics of the students (learning styles, needs, interests, etc.), have allowed the creation of paths and personalized learning with intelligent tutoring systems that direct the student toward educational content based on his profile and offer personalized feedback and assessments (Wang et al., 2022). Personalized online educational spaces support better learning outcomes (Walkington and Bernacki, 2019). For example, AI tools can improve the learning experience in language studies compared to “human peers” (Fryer et al., 2017).

Pegaso Telematic University, as one of world-leaders in distance learning and the use of new technologies for education, is currently evaluating AI solutions to improve its educational programs as well as the platform that is already used by students and professors to support learning. Our assessment of some opportunities available on the market has led us to share reflections that resulted in the present contribution.

As hinted at above, conversational pedagogical agents available for education still tend to focus on specific tasks: for example, it is possible to create chatbot-based solutions that are trained in course materials and can respond to students’ questions anywhere, anytime. Most of these areas regard the application of AI in reproducing teaching tasks and supporting learning by acting as a lecturer. A crucial aspect of AI is the ability to adapt the contents based on the learner’s characteristics and needs that could emerge during the learning process. According to this view, the teacher, who would be able to exercise empathy, could perform the adaptation. Nevertheless, the AI tools have a great asset: the ability to reduce the time delay in the reply. This is even more relevant because the importance of e-learning increased because of the COVID-19 pandemic (Lemay et al., 2021; Kim et al., 2022).

It is possible to identify five strength points in a system able to provide intelligent, personalized support promptly. The actions that make the conversational pedagogical agent a desirable educational resource are:

1. Providing immediate feedback, e.g., timely corrections to the student’s answers;
2. Aligning the replies with the lesson progression; in other words, a chatbot could respond to questions considering the course material that the student is accessing in the here-and-now or is about to in the next sections;
3. Maintaining the student in the flow experience in a personalized manner; indeed, not being able to receive a response while studying may lead to a “break in presence” in the usage of technology that would negatively affect recall and the learning experience as a whole (Ahn et al., 2022);
4. Preventing the rise of doubts or misunderstandings on fundamental concepts while the study process is being carried on;
5. Providing information about prerequisites needed for a specific lesson or course material that the student is about to access, possibly based on information about the individual student’s progress;
6. Stimulate critical thinking through the promotion of conversational-based learning, which is a learning mode that emerges from an exchange of questions and answers between chatbot and student (Rospigliosi, 2023).

Therefore, while the timeliness of “basic” chatbots (i.e., conversational pedagogical agents trained to only answer questions about course materials) could be an asset, it is possible to extend their capabilities and identify lines of improvement. The primary challenge associated with the use of chatbots pertains to achieving genuine personalization in learning and seamless integration into various virtual learning environments. In distance education communication barriers, lack of interactions, and difficulty in obtaining immediate feedback from teachers are significant drawbacks (Zaheer and Munir, 2020; Kusmaryono et al., 2021). The interactivity of chatbots plays a crucial role in distance learning, with increasing utilization observed in addressing engagement in higher education institutions (Studente and Ellis, 2020; Wollny et al., 2021), thereby fostering a motivating environment even in online and distance learning contexts (Chumkaew, 2023). Chatbots in distance learning cover two primary functions: providing support and delivering feedback (Vázquez-Cano et al., 2021). Consequently, we have identified four specific use case scenarios on the basis of these main functions (see Table 1) in which conversational pedagogical agents can support the work and role of professors/educators, with particular attention to distance education according to a human-in-the-loop approach.

**Table 1:** Use case scenarios for AI tools that support higher distance education.

Use case scenarios	Challenge	What AI systems would/could do	Human-in-the-loop	Outcomes/Mesurable indicators	Examples from the literature
Providing support to the student about the course materials	Distance education universities welcome a large number of students. Distance learning, however, does not allow direct contact between student and professor during each lesson, and students often follow recorded lessons asynchronously; for this reason, if the student has doubts or questions regarding the teaching material, he/she can contact the professor privately with a highly variable response time. The large number of students in distance education institutions could represent notable additional workload for the professor	As it has been experimented in our institution, an AI tool can be trained on the contents of a specific course, so that a chatbot can immediately respond to students’ questions about the contents of the course, decreasing the teachers’ overload in distance teaching	The professor designs the course materials. Moreover, the professor could review and improve the chatbot’s response by adding updated materials, links to additional learning resources for in-depth analysis, and also obtain information on students’ understanding of course contents	Immediate assessment of students’ understanding and learning outcomes after interaction with the chatbot (vs. control groups without it); Students’ satisfaction level measured after interaction with the chatbot	Clarizia et al. (2018)—*example of a Chatbot prototype that starting support the students with answers;* Chen et al. (2023)—*empirical study about a chatbot intelligent student assistants that shows the effectiveness in teaching new concepts* Liu et al. (2022)—*analysis of a chatbot based on inquiry evaluation aimed to increase students’ satisfaction level* Lee et al. (2020)—*chatbot as online tutor to reduce teachers workload in distance learning* Wollny et al., (2021)*—systematic review that shows three categories for mentoring chatbots (scaffolding, recommending, informing)*
Thesis management	The high number of students in distance education universities implies an important number of theses and student papers that professors must check every semester	AI could support professors in managing theses by taking care of a preliminary evaluation of the content and especially of formal aspects and adherence to guidelines, plagiarism control and verification of correct citation of sources	The professor can focus on evaluating the content of the theses and offer specific indications to the student for its improvement	Decreased time devoted to student thesis evaluation after implementation of the tool; Professors’ satisfaction level measured after utilization of the tool for thesis evaluation;	*There is literature on chatbots used to aid thesis writing, like* Malik et al., (2023), Schwenke et al. (2023)*, but we did not find works on AI tools used to support the management of high numbers of students’ theses or essays* Schwenke et al. (2023)*—solutions about writing bachelor thesis supported by chatbots* Malik et al. (2023) *study on perception of students about AI-powered writing tools in academic essay*
Providing support to the student on administrative tasks in their university career	Distance education institutions are typically characterized by sophisticated online platforms that support the learning activities, and the utilization of administrative features such as enrolling in courses and exams and accessing multiple online services	AI tools and chatbots can support students and administrative workers in utilization of the platform and, based on individual profiling and data training, anticipate their needs, and facilitate interaction with complex platforms (e.g.: reminding deadlines based on individual education plan. Managing admission process)	Administrative personnel intervene when issues arise that cannot be managed by AI tools. Furthermore, they have immediate access to individual students’ information and their needs	Reduced time of administrative efforts; Reduced number of complaints/request for direct support to administrative offices after implementation of the tool; Students’ satisfaction level measured after interaction with the chatbot;	Košecká and Balco (2023)—*identification of critical paths in the application of chatbots in the administrative tasks* Košecka et al. (2022)—*evaluation of implementation of chatbots for the work productivity in general terms* Lee et al. (2019)—*empirical study on the reduction of the administrative workload using a chatbot for students’ FAQs* Lee et al. (2020)—*solution that manages the class schedule and the student profiles* El Hefny et al. (2021)*—a prototype of a chatbot developed for the improvement of admission process in universities*
Online classes with personalized educational contents; student-centered learning aids and tools	Not all students have the same characteristics in terms of learning styles, needs, interests, etc. Therefore, not all educational content is suitable for all learners. Distance education is also supposed to meet specific needs such as work-study integration	AI tools could analyze students’ characteristics in order to sort them into specific online classes with educational content suited to their needs, and/or point them toward specific learning aids tailored on particular situations with specific and personalized feedback	The professor could focus on creating different teaching materials for each course content suitable for specific types of users, and develop learning aids that respond to students’ necessities	Students’ evaluation of course contents after personalization (compared with previous non-personalized learning)	Kaiss et al., (2023)—*description of a prototype integrated in Moodle platform providing personalized learning objects in distance learning* Wu et al. (2020)—*meta-analysis that shows the effect on students’ learning outcomes, personalizing the path and alleviating learners’ anxiety* El Janati et al. (2020)—*proposal of an adaptive chatbot that reduce response time* Wollny et al. (2021)*—systematic review that provides six publications that focus on the adaptation of the chatbot on the student’s needs.*

The implementation of the scenarios proposed in Table 1 involves a preliminary collection of data on the profile of students who use chatbots in the field of e-learning (Wu et al., 2020). In particular, the possibility to realize these scenarios requires a profiling of students based on different types of information. These can be collected, for example, in the form of demographic data, school achievement data and psychological data (e.g., questionnaires; behavioral measures such as frequency and duration of access to services) in order to define needs and specific learning styles so that personalized content could be designed (Vladova et al., 2019; Kaiss et al., 2023).

It should be emphasized that conversational pedagogical agents should not replace teachers and professors in any aspect of the learning processes but rather interact with them in a collaborative process as the capabilities of humans and AI can be complementary (Chen F., 2022; Chen Z., 2022). While humans outperform AI in dealing with new and unstructured problems where emotional and value communication as well as adaptation to the environment are necessary, AI outperforms humans in addressing repetitive, objective problems, structured ones that require data management. In this sense, the cooperation between teachers and artificial educational agents, represents the future of education. In this framework, it is essential to also consider the ethical implications. When conversational chatbots handle sensitive data, such as managing students’ academic records, adhering to regulations like the EU’s General Data Protection Regulation (GDPR) becomes crucial for guiding automated decision-making processes that may arise. Additionally, clear communication to the users regarding the disclosure of conversations with chatbots is crucial (Okonkwo and Ade-Ibijola, 2021). Furthermore, in all scenarios proposed where conversational agents serve in the personalization of the learning experience is pivotal the communication to the users about the nature of the interaction as non-human to prevent misinterpretations (Adamopoulou and Moussiades, 2020). As hinted at above, also in education it is necessary to implement a “human-in-the-loop” approach (Mosqueira-Rey et al., 2023), namely prefiguring and defining the role of humans within a process that is just partially automatized. In particular, the teacher can support and coordinate AI in tackling open teaching problems and, supported by technology in some tasks, use creative energies to improve students’ learning motivation as well as the quality of the relationship with them (Tong et al., 2019). Furthermore, according to a vision of Usable artificial intelligence (Xu, 2019) the results of AI must be accessible to users for real collaborative learning between man and machine to take place.

## 5 Discussion

### 5.1 “Better than my professor”?

To position themselves as a truly groundbreaking technology in higher education, conversational pedagogical agents should be able to do something more than a human professor. As we will see later in the present contribution, this does not mean in any way that we expect that any AI tool could fully take on the role of teachers and educators and replace them. Yet, AI tools and especially conversational pedagogical agents would be able to do something more than just respond earlier than a human to students’ questions. Like what doctor Topol (2019) said about the implementation of AI in healthcare, this technology would be as useful in education as it could carry out technical and bureaucratic tasks, leaving more space and time to human professionals (here, the professors) to interact with students and properly respond to their educational needs.

In other words, while the scenarios described in Table 1 already defined advanced implementation examples of AI tools in distance higher institutions’ educational efforts, we think it is important to prefigure additional ideas that could drive the design of innovative AIED for distance higher education. While these ideas still relate to Table 1 scenarios in terms of general aims (i.e., supporting course fruition and improving personalization of learning), they make some steps further toward contents and features of the learning process that go beyond the mere course materials. For example, AI (and especially conversational pedagogical agents supported by sophisticated data analysis) implemented in high-level education could perform the subsequent tasks:

- Collecting user data from the interaction with the students and classifying their necessities; e.g., finding specific information within course material vs. simulating examination vs. suggesting links, additional materials for in-depth analysis beyond course materials;
- Providing personalized suggestions about studying *methods* and about how to approach the whole study program;
- Identifying and highlighting connections between study materials from different courses; designing conceptual maps, or analyzing, correcting and improving students’ notes;
- Facilitating the communication between the student and the professor by providing automated responses to simple questions (e.g., reminding deadlines and formal procedures) while forwarding to the professor the questions related to scientific content only;
- Drawing from behavioral change and motivation theories to provide adapted motivational feedback (suggestions, encouragement, even hedonic comments and humor) to keep students’ engagement at a high level; this could be tailored to students’ personality as well as communicational style and explicit needs. Indeed, personalized and specific motivational feedback (instead of generalized nudges or encouragement) is proven to improve learning outcomes, Perikos et al., 2017).

Such a chatbot would actually exceed the mere “teaching,” crossing over to authentic educational effort. Of course, when we prefigure such a technology, it is more and more important to recover the role of human educators. AI modules can help the teacher ensure a personalized approach and ongoing support that may not be feasible for a human teacher. Previous studies have demonstrated the positive impact of chatbot applications on learning outcomes, although these effects may vary depending on instructional levels and the duration of intervention in the learning application (Wu and Yu, 2023). However, the presence of human lecturers remains unquestionably important. Human professors play a crucial role in addressing complex questions that extend beyond the scope of a single course, requiring multidisciplinary and multifaceted knowledge. Only a human lecturer can guide students when critical judgment or ethical considerations are needed. The strength of AI chatbot applications lies in easing the burden on lecturers, allowing them to avoid being overwhelmed by a flood of emails with short, delayed responses. Instead, AI chatbots can enhance interactions with students through dedicated synchronous, even online, lessons that encourage meaningful engagement after students have had time for in-depth reflection.

## 6 Conclusion

The present contribution resumed the opportunities represented by AI, especially in the field of distance higher education, focusing mainly on AI-based tools that could interact with learners directly to support technology-mediated learning processes (conversational pedagogical agents and chatbots). Based on the literature and our reflections while evaluating resources for possible integration into our institution’s educational programs, we have identified the positive aspects of chatbots being able to respond in a timely manner, but also maintained that an AI system for learning should be able to do “something more” than what a human professor could do just with less punctuality. As we have based our considerations on a purpose-oriented literature search that does not constitute a comprehensive representation of the field, future studies may include systematic review and meta-analytic efforts to explore the effects of innovative features of conversational pedagogical agents on learning outcomes, with a focus on distance education. More generally, future research could test advanced AI-based solutions, considering the importance of not replacing human educators but offering actual, desirable support to study activities and the quality of the learning experience.

## Data availability statement

The original contributions presented in the study are included in the article/supplementary material, further inquiries can be directed to the corresponding authors.

## Author contributions

ST: Conceptualization, Writing – original draft, Writing – review & editing. RF: Writing – original draft, Writing – review & editing. CS: Investigation, Writing – review & editing. EM: Investigation, Writing – review & editing. PL: Supervision, Writing – review & editing.
